# Understanding Health-related quality of life in rheumatologic diseases: insights from PROMIS^®^ health domains

**DOI:** 10.1186/s41687-026-01138-6

**Published:** 2026-07-10

**Authors:** Milan Kock, Christoph Paul Klapproth, Annika Döhmen, Jonas Prenißl, Felix Fischer, Udo Schneider, Matthias Rose, Alexander Obbarius

**Affiliations:** 1 Center for Patient-Centered Outcomes Research, Department of Psychosomatic Medicine, Center for Internal Medicine and Dermatology, Charité – Universitätsmedizin Berlin, Berlin, Germany; 2 Berlin Institute of Health at Charité – Universitätsmedizin Berlin, BIH Biomedical Innovation Academy, BIH Charité Digital Clinician Scientist Program, Berlin, Germany; 3 Department of Paediatrics, University Hospital Brandenburg an der Havel, Brandenburg Medical School, Brandenburg an der Havel, Germany; 4 Department of Rheumatology and Clinical Immunology, Center for Internal Medicine and Dermatology, Charité – Universitätsmedizin Berlin, Berlin, Germany; 5 Department of Quantitative Health Sciences, Medical School, University of Massachusetts, Worcester, MA, USA; 6 Dornsife Center for Self-Report Science, University of Southern California, Los Angeles, CA USA

**Keywords:** Patient reported outcome (PRO), PROMIS^®^ (Patient-Reported Outcomes Measurement Information System^®^), Health related Quality of Life HRQoL, EQ5D5L questionnaire, Rheumatologic disease

## Abstract

**Background:**

Rheumatologic diseases significantly impact health-related quality of life (HRQoL), affecting physical, mental, and social well-being and pose unique challenges due to their wide range of symptoms. Patient-reported outcomes (PROs) provide valuable insights into how these diseases influence different dimensions of HRQoL, supporting personalized healthcare approaches.

**Methodology:**

In this cross-sectional study, we examined HRQoL in 213 patients with rheumatologic diseases, including, among others, rheumatoid arthritis, localized pain disorders, connective tissue diseases, and rarer conditions such as vasculitis, using the EQ5D-5L and the Patient-Reported Outcomes Measurement Information System (PROMIS^®^) as well as Pain and HRQoL specific PROs. We provided PROMIS health domain scores of key health aspects such as physical function, fatigue, pain interference, and social participation. Additionally, we compared HRQoL and PROMIS domain scores with a German general population reference sample and conducted exploratory comparisons across the three most frequent principal diagnosis groups: systemic sclerosis, rheumatoid arthritis, and vasculitis. Moreover, we investigated which patient-reported health domains were most strongly associated with HRQoL in these diseases. Penalized regression models with elastic net regularization were used to identify stable predictors of HRQoL, followed by a pooled linear regression model for interpretation.

**Results:**

Individuals with rheumatic diseases had significantly lower HRQoL than the general population (EQ-5D-5L VAS mean: 55.4 versus 73.2). PROMIS T-scores indicated markedly reduced Physical Function (M = 36.5, SD = 9.6) and elevated pain interference (M = 61.7, SD = 9.2). Penalized regression analyses identified Physical Function, Fatigue, Ability to Participate in Social Roles and Activities as stable predictors of EQ-5D-5L VAS. Higher Physical Function and Social Participation were associated with higher HRQoL, whereas higher Fatigue was associated with lower HRQoL.

**Conclusions:**

These results highlight the importance of PROs in understanding the patient experience in rheumatologic disease and identifying individual needs. Routine assessment of HRQoL domains may help recognize unmet needs and support individualized care in rheumatology.

**Supplementary Information:**

The online version contains supplementary material available at 10.1186/s41687-026-01138-6.

## Background

Patient-Reported Outcomes (PROs), particularly those assessing Health-related Quality of Life (HRQoL), have become central to understanding and managing chronic diseases across various medical disciplines [1]. Collecting HRQoL in clinical trials has led to a better understanding of how diseases impact patients’ health [2–4]. At the same time, routine measurement of HRQoL in clinical care can support shared decision-making, guide treatment adaptations, and track the progression or remission of symptoms [5].

In rheumatology, the importance of PROs has long been recognized. Many symptoms of rheumatic diseases, such as pain, fatigue, or impaired physical function, are best captured through patient self-report [6]. Numerous studies have demonstrated that HRQoL is reduced across rheumatologic diseases [7–14]. For example, in rheumatoid arthritis, both physical and mental health domains are often negatively affected [7, 15]. In vasculitis, fatigue and reduced social participation have emerged as key drivers of impaired HRQoL [9, 16, 17]. In systemic sclerosis, physical limitations are particularly prominent [8], whereas patients with systemic lupus erythematosus (SLE) frequently report fatigue as a dominant symptom affecting both physical and emotional well-being [18–21]. Despite its relevance, fatigue remains inconsistently addressed in clinical management [22].The European Alliance of Associations for Rheumatology (EULAR) has therefore emphasized the need for a stronger focus on biopsychosocial health dimensions and self-management strategies in routine care [23, 24].

A growing number of tools are available to assess HRQoL in rheumatology, yet many studies still rely on legacy instruments [9, 11, 16, 19, 25]. The Patient-Reported Outcomes Measurement Information System^®^ (PROMIS^®^), developed with support from the U.S. National Institutes of Health, provides a flexible and psychometrically robust set of instruments to assess key health domains. PROMIS instruments use item response theory (IRT), allow the use of short forms or computerized adaptive testing (CAT), and are increasingly adopted in international research and clinical initiatives [26, 27].

In summary, while there is consistent evidence that HRQoL is significantly impaired in individuals with rheumatologic diseases, there is limited knowledge about which health domains most strongly predict overall quality of life. Identifying the most relevant PRO domains can help guide targeted, patient-centered interventions. Therefore, the present study aimed to: (1) describe core self-reported health domains in a university hospital sample of patients with rheumatologic diseases; (2) compare HRQoL and PROMIS domain scores with a German general population reference sample and, exploratorily, across the three most frequent principal diagnosis groups; and (3) identify sociodemographic, clinical, and patient-reported health domains associated with overall HRQoL.

## Methods

### Sample

We collected cross-sectional data from patients during their inpatient stay at the Department of Rheumatology and Clinical Immunology at Charité – University Medical Center Berlin between September 2018 and August 2019 via convenience sampling. To minimize selection bias, all eligible inpatients treated during the recruitment period were invited to participate. Patients were excluded if they had insufficient language skills or had cognitive impairment. One patient with fever of unknown origin was excluded from the present analyses because this diagnosis did not represent a rheumatologic condition. For exploratory diagnosis-specific comparisons, we selected the three most frequent principal diagnosis groups in the sample, defined using principal ICD-10-GM codes: systemic sclerosis (SSc; M34.0 and M34.8), rheumatoid arthritis (RA; M06.99), and vasculitis (I77.6 and M31.3). “+” suffixes were pooled with their corresponding base codes. The reference data used in this study originated from a study that compared norm values of the PROMIS-29 Profile in three countries including in Germany [28].

### Measures

Participants completed a questionnaire battery that included sociodemographic (age, gender, living status, educational level, work status) and clinical characteristics (pain duration, frequency of pain attacks, number of diagnoses, pain therapy during the last year), as well as PROMIS health domains, a pain-specific PROM, and the EQ-5D-5L as a measure of HRQoL.

Nine different PROMIS domains were assessed. While pain interference was assessed by the whole item bank (v1.1, 40 items), short-forms were used for the assessment of all other domains: Pain intensity Scale v1.0 (3 items), physical function v2.0 customized short-form (23 items), fatigue v1.0 customized short-form (6 items), depression v1.0 customized short-form (6 items), anxiety v1.0 short-form 4a (4 items), sleep disturbance v1.0 customized short-form (6 items), Ability to Participate in Social Roles and Activities v2.0 customized short-form (6 items). For each domain a T-score was calculated, using the HealthMeasures Scoring Service (https://www.healthmeasures.net). On this metric, a score of 50 represents the mean of the general population and the standard deviation is 10. Higher T-scores are equivalent to a higher expression of the underlying construct (for example better physical function, higher Ability to participate in social activities or more depression/ anxiety/ fatigue/ sleep disturbance).

The Regional pain scale (RPS) was used to assess pain dissemination across the body with 19 items. Item scores range between 0 and 3, higher scores indicate greater dissemination [29].

Health- related Quality of life (HRQoL) was measured with the EQ-5D-5L which includes a descriptive part and a visual analogue scale (VAS) [30]. The descriptive part includes the five dimensions mobility, self – care, usual activities, pain/ discomfort, and anxiety/ depression. Each dimension is scored individually by patients on one of five levels. Those levels range from “no problems” to “extreme problems.” The responses to each of these dimensions are then mapped to the patient’s respective health state. In addition, the visual analogue scale allows patients to plot their perceived health on a vertical axis with a range of values from 0 to 100. The respective endpoints of the axis are labeled as best or worst health imaginable. Furthermore, decision experiments determined which health states subjects would prefer over another. In the subsequent scoring, a value was assigned to each different health state of the EQ-5D, with 1 describing the best health state, 0 describing death, and negative values describing states worse than death [31]. In this study we measured HRQoL via VAS and the EQ-5D-5L index value for which we used the German value set by Greiner et al. [32].

### Statistical analysis

Statistical analyses were performed using IBM SPSS Statistics version 27 [33] and R version 4.5.2 [34]. Descriptive statistics were used to present clinical and sociodemographic characteristics. Continuous data were presented as mean ± SD, while categorical data was expressed as absolute and relative frequencies. All quantitative variables were analyzed as continuous variables. Differences in overall HRQoL (EQ-5D-5L VAS, and EQ-5D-5L index value) and PROMIS score mean values of the rheumatology sample and reference population were calculated with independent samples t-tests. Differences between patients with SSc, RA, and vasculitis were examined using one-way analysis of variance (ANOVA) for EQ-5D-5L VAS and PROMIS domain scores. Tukey HSD post-hoc tests were conducted following statistically significant omnibus tests. Spearman correlation coefficients were used to assess bivariate relationships between the different predictors. Correlation coefficients were interpreted based on their absolute values, classified as small for values between 0.1 and 0.2, medium for 0.3–0.4 and large for ≥ 0.5, with 0 indicating no correlation [35].

To identify predictors of HRQoL, the EQ-5D-5L VAS was selected as the dependent variable because it provides a direct global, patient-reported assessment of perceived overall health, while remaining conceptually distinct from the explanatory variables used. All prespecified predictors (sociodemographic, clinical, and patient-reported outcomes) were entered simultaneously into regression models. Derived variables included the number of diagnoses (based on all available ICD codes) and a summary measure of pain-related therapies in the previous year.

To reduce overfitting and improve the stability of predictor selection, penalized regression models were applied using elastic net regularization as implemented in the glmnet package (mixing parameter α = 0.5) [36]. Categorical predictors were represented by indicator variables, and predictor variables were internally standardized during penalized model estimation. Model selection was based on 5-fold cross-validation using a predefined grid of penalty parameters. Both the minimum cross-validated error (λ_min) and the more parsimonious 1-standard-error criterion (λ_1se) were evaluated, with λ_1se selected as the primary model. Predictor importance was assessed based on selection frequency across imputed datasets. Model performance was summarized using cross-validated mean squared error (MSE) and the number of selected predictors. For descriptive purposes, mean penalized coefficient estimates and their standard deviations were calculated across imputations.

Prior to imputation, the proportion of missing values was summarized for each analysis variable. Missing data were assumed to be missing at random (MAR) and handled using multiple imputation by chained equations with the mice package [37]. Twenty imputed datasets were generated using predictive mean matching for continuous variables and logistic regression for binary variables, while variables without missing values were not imputed. Penalized regression models were fitted separately within each imputed dataset, and results were summarized across imputations by calculating selection frequencies and average coefficient estimates. Convergence of the imputation procedure was assessed using trace plots of chain means and variances.

The primary analysis was based on multiply imputed data. Complete-case elastic net analyses were conducted as sensitivity analyses. In addition, lasso regression models (α = 1) were estimated to evaluate the stability of predictor selection under alternative regularization. To facilitate interpretation, a standard linear regression model including predictors selected in 100% of the imputed datasets under the primary elastic net λ_1se criterion was then estimated across the imputed datasets, and regression coefficients were pooled using Rubin’s rules [38]. This final linear regression model was estimated using the original, non-standardized variable scales; therefore, coefficients are reported as unstandardized regression coefficients.

## Results

### Patient characteristics

This study included 213 patients with rheumatologic conditions. Patients were predominantly female (*n* = 144; 67.6%) and had a mean age of 55.7 (SD = 16.9) years. These and other patient characteristics are reported in Table 1.

**Table 1:** Participant characteristics of the rheumatology sample (*n* = 213)

**Age in years, mean ± SD (range)**	55.7 ± 16.9 (19.2–88.6)
**Gender, n (%)**	
Female	144 (67.6)
Male	69 (32.4)
**Living status**,** n (%)**	
With partner	134 (62.9)
Single	63 (29.6)
In Care	1 (0.5)
Other	6 (2.8)
Unknown	9 (4.2)
**Educational level**,** n (%)**	
Doctoral or Equivalent	5 (2.3)
Bachelor’s / Master’s degree or equivalent	73 (34.3)
Degree of post-secondary/ tertiary education	83 (39.0)
Degree of secondary education	36 (16.9)
Degree of primary Education	2 (0.9)
Without	3 (1.4)
Unknown	11 (5.2)
**Work status**,** n (%)**	
Full-time	29 (13.6)
Part-time	21 (9.9)
Seeking employment	3 (1.4)
Not employed (student, retired, freelancer)	79 (37.1)
Unable to work (due to pain)	30 (14.1)
Unable to work (due to other reasons)	30 (14.1)
Unknown	20 (9.4)
**Most frequent rheumatologic conditions**,	
**n (%)**	
SSc	51 (23.9)
RA	20 (9.4)
Vasculitis	22 (10.3)
Other	120 (56.3)
**Number of Diagnoses**,** mean ± SD (range)**	8.9 ± 5.6
	(1.0–32.0)
**Pain Duration**,** n %**	
< 1 month	10 (4.7)
1–6 months	24 (11.3)
6–12 months	14 (6.6)
1–2 years	21 (9.9)
2–5 years	37 (17.4)
> 5 years	83 (39.0)
Unknown	24 (11.3)
**Pain Therapy in the last year**,** n (%)**	
Yes	170 (79.8)
No	19 (8.9)
Unknown	24 (11.3)
**Frequency of Pain Attacks**,** n (%)**	
Daily	72 (36.6)
Several times per week	61 (30.8)
Once per week	6 (2.8)
Less than once per week	26 (12.1)
Never	33 (15.4)
Unknown	16 (7.5)
**Regional Pain Scale sum score**,** mean ± SD (range)**	14.1 ± 10.9 (0.0–54.0)

n = Number; SD = Standard deviation; M = Mean

### Differences between the rheumatology sample and reference population sample

**Fig. 1:**
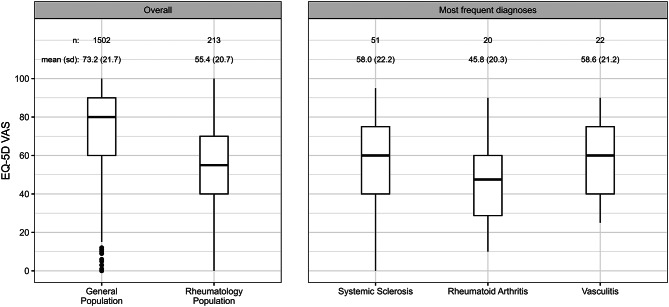
Boxplots of EQ-5D-5L VAS scores in the German general population reference sample, the total rheumatology sample, and the three exploratory diagnosis-specific groups: systemic sclerosis, rheumatoid arthritis, and vasculitis. Boxes span the interquartile range (IQR); central line: median; whiskers extend to 1.5x IQR; points beyond whiskers indicate outliers; n: number; sd standard deviation

Table 2 shows PRO scores from the rheumatology sample and the general population. The weighted reference population sample consisted of a group of 1502 German participants with a mean age of 50 years, and a somewhat lower proportion of women (*n* = 773.1; 51.5%). PROMIS T-Scores have been standardized based on the general population in the United States, thus values of the German reference population sample are slightly different from 50. The mean EQ-5D-5L VAS score of the reference population sample was 73.2 (SD = 21.7). Patients in the rheumatology sample reported a mean EQ-5D-5L VAS score of 55.4 (SD = 20.7), compared with 73.2 (SD = 21.7) in the German general population reference sample. The mean difference was 17.8 points, 95% CI [14.7, 20.9], *p* < 0.001, corresponding to a large standardized difference (d = 0.8). In the diagnosis-specific comparison, EQ-5D-5L VAS scores did not differ significantly between patients with systemic sclerosis, rheumatoid arthritis, and vasculitis (Table 3; Fig. 1). However, EQ-5D-5L VAS scores showed wide ranges within each group, ranging from 0 to 95 in SSc, 10 to 90 in RA, and 25 to 90 in vasculitis. Among the PROMIS domains, only pain interference differed significantly between diagnosis groups, with higher scores in patients with rheumatoid arthritis than in patients with systemic sclerosis.

**Table 2:** PROMIS T-Scores and EQ-5D-5L values of the rheumatology sample and reference population sample

Instrument scores, M (SD, range)			t-Test	
Predictors	Rheumatology group	Reference population	t (df)	*p*-value
Pain interference	61.7 (9.2, 37.3–83.0)	51.3 (9.6, 41.6–75.6)	15.4 (281.4)	< 0.001
Physical function	36.5 (9.6, 12.0–60.1)	51.2 (7.8, 22.9–56.9)	-25.1 (1713.0)	< 0.001
Sleep disturbance	53.9 (9.1, 29.6–73.4)	49.5 (8.9, 32.0–73.3)	6.8 (1710.0)	< 0.001
Ability to participate in Social Roles and Activities	43.8 (7.5, 27.5–64.2)	51.1 (8.9, 27.5–64.2)	-12.9 (299.9)	< 0.001
Anxiety	54.5 (9.3, 40.3–81.4)	52.5 (8.5, 40.3–81.4)	3.14 (1709)	0.002
Depression	56.0 (9.0, 38.2–79.3)	52.0 (8.7, 41.0–79.3)	6.3 (1713.0)	< 0.001
Fatigue	56.2 (9.7, 33.2–76.6)	48.3 (10.2, 33.7–75.8)	10.5 (1712.0)	< 0.001
EQ-5D-5L VAS	55.4 (20.7, 0–100)	73.2 (21.7, 0–100)	-11.1 (1707.0)	< 0.001
EQ-5D-5L index value	0.7 (0.3, -0.7–1.0)	0.9 (0.2, -0.1–1.0)	-10.1 (217.0)	< 0.001

M = Mean; SD = Standard deviation; df = degrees of freedom, t = t-Test statistic

**Table 3:** PROMIS T- Scores and EQ-5D-5L values of ICD-based diagnosis groups

Instrument scores, *n*, M (SD)				ANOVA	
Predictors	Systemic sclerosis (SSc)	Rheumatoid arthritis (RA)	Vasculitis	F (df1, df2)	*p*-value
Pain interference	51, 59.5 (10.0)	20, 66.4 (6.0)	22, 60.3 (12.0)	3.61 (2, 90)	**0.031**
Physical function	51, 35.3 (9.6)	20, 31.5 (8.4)	22, 38.5 (10.0)	2.89 (2, 90)	0.061
Sleep disturbance	50, 54.6 (8.5)	20, 54.7 (9.8)	22, 51.3 (12.1)	0.97 (2, 89)	0.384
Ability to participate in Social Roles and Activities	51, 45.3 (7.8)	20, 41.1 (7.5)	22, 45.0 (7.9)	2.19 (2, 90)	0.117
Anxiety	51, 53.8 (9.1)	19, 57.5 (10.0)	22, 54.8 (10.8)	0.99 (2, 89)	0.375
Depression	51, 54.9 (8.5)	20, 58.9 (8.8)	22, 54.2 (11.6)	1.59 (2, 90)	0.209
Fatigue	50, 55.6 (8.2)	20, 59.4 (9.2)	22, 56.2 (12.2)	1.17 (2, 89)	0.315
EQ-5D-5L VAS	49, 58.0 (22.2)	20, 45.8 (20.3)	21, 58.6 (21.2)	2.60 (2, 87)	0.080

n = Number; SD = Standard deviation; M = Mean; F = F-statistic from a one-way ANOVA; df = degrees of freedom (df1 = between groups; df2 = within groups); Groups (ICD-codes):SSc = M34.0, M34.8; RA = M06.99; Vasculitis = I77.6, M31.3

### Correlational analyses

Correlations between the main clinical and patient-reported variables are provided in Table 4. Noteworthy was the high correlation between Anxiety and Depression (ρ = 0.780). Among all predictors, pain duration showed the weakest significant correlation with the EQ-5D-5L VAS (ρ = -0.156).

**Table 4:** Correlational analyses between predictors of HrQoL

	Variable	1	2	3	4	5	6	7	8	9	10	11	12	13	14
1	Pain Duration	--													
2	Frequency of Pain Attacks	0.015	--												
3	Number of Diagnoses	0.207**	− 0.100	--											
4	Pain Therapy last year	0.014	− 0.317**	− 0.114	--										
5	Sum of RPS	0.247**	− 0.399**	0.238**	0.245**	--									
6	Pain Interference	0.079	− 0.469**	0.133	0.173*	0.581**	--								
7	Physical Function	− 0.229**	0.301**	− 0.356**	− 0.028	− 0.384**	− 0.556**	--							
8	Sleep Disturbance	0.032	− 0.348**	− 0.004	0.155*	0.419**	0.513**	− 0.271**	--						
9	Ability to Participate in Social Roles and Activities	− 0.050	0.290**	− 0.154*	− 0.240**	− 0.356**	− 0.595**	0.529**	− 0.273**	--					
10	Anxiety	0.125	− 0.207**	0.042	0.213**	0.324**	0.486**	− 0.269**	0.408**	− 0.306**	--				
11	Depression	0.068	− 0.283**	0.075	0.157*	0.295**	0.550**	− 0.296**	0.457**	− 0.331**	0.780**	--			
12	Fatigue	0.164*	− 0.325**	0.149*	0.178**	0.440**	0.615**	− 0.448**	0.516**	− 0.503**	0.579**	0.616**	--		
13	EQ-5D-5L VAS	− 0.156*	0.304**	− 0.263**	− 0.041	− 0.336**	− 0.452**	0.476**	− 0.267**	0.450**	− 0.267**	− 0.351**	− 0.478**	--	
14	EQ-5D-5L index value	− 0.099	0.424**	− 0.251**	− 0.155*	− 0.480**	− 0.656**	0.695**	− 0.333**	0.560**	− 0.448**	− 0.479**	− 0.568**	0.588**	--

*Correlation is significant at the 0.05 level (2-tailed)

**Correlation is significant at the 0.01 level (2-tailed)

### Predictors of health-related quality of life

The proportion of missing data was generally low across variables, with the highest proportion observed for pain duration (11.3%) and frequency of pain attacks (7.5%) (Supplementary Table S1).

Penalized regression analyses based on multiply imputed data identified a small set of stable predictors of HRQoL (Table 5). Physical Function, Fatigue, and Ability to Participate in Social Roles and Activities were selected in 100% of imputed datasets, indicating highly stable associations across imputations. Frequency of pain attacks was selected in 70% of models. All other predictors showed low selection frequencies, including Depression and Regional Pain Scale (20% each), Pain Interference (15%), and Age (5%).

**Table 5:** Selection frequency of predictors of HRQoL^1^

Predictor	Selection frequency (%)	Selected in complete-case analysis
Social Participation	100	Yes
Fatigue	100	Yes
Physical Function	100	Yes
Frequency of pain attacks	70	No
Depression	20	No
Regional Pain Scale	20	No
Pain Interference	15	No
Age	5	No

^1^Selection frequency based on elastic net models (α = 0.5) across 20 imputed datasets using the 1-standard-error rule

Using the minimum-error criterion (λ_min), a larger set of predictors was selected. In addition to the three core predictors, Depression, Age, Frequency of pain attacks, and Number of diagnoses were more frequently retained, although with lower stability compared to the λ_1se model.

Average penalized coefficient estimates across imputations are presented in Supplementary Table S3. The direction of coefficients was consistent with the selection frequency results, with positive coefficients for physical function and social participation, and a negative coefficient for fatigue. Coefficients for other predictors were small and unstable.

In the pooled linear regression model (Table 6), Physical Function (B = 0.602, SE = 0.148, 95% CI 0.309 to 0.894, *p* < 0.001), Social Participation (B = 0.558, SE = 0.209, 95% CI 0.144 to 0.972, *p* = 0.009), and Fatigue (B = − 0.500, SE = 0.156, 95% CI − 0.807 to − 0.192, *p* = 0.002) were independently associated with EQ-5D-5L VAS. Based on the pooled linear regression model, the predicted EQ-5D-5L VAS can be approximated as:

EQ-5D VAS ≈ 36.93 + 0.60 × Physical Function + 0.56 × Social Participation − 0.50 × Fatigue

**Table 6:** Final pooled linear regression model for EQ-5D-5L VAS^1,2^

Predictor	B	SE	95% CI	*p*-value
Constant	36.929	15.601	6.051 to 67.807	0.019
Physical Function	0.602	0.148	0.309 to 0.894	< 0.001
Social Participation	0.558	0.209	0.144 to 0.972	0.009
Fatigue	-0.500	0.156	-0.807 to -0.192	0.002

B = unstandardized regression coefficient; SE = standard error; CI = confidence interval

^1^Regression coefficients were estimated in a standard linear regression model across 20 multiply imputed datasets and pooled using Rubin’s rules.

^2^Predictors were selected based on the elastic net model using the 1-standard-error criterion.

Sensitivity analyses supported the robustness of the main findings. Across modeling approaches, the three core predictors - Physical Function, Fatigue, and Social Participation - were consistently selected in both imputed and complete-case analyses. Differences between models were limited to less stable predictors. In the complete-case analysis, Anxiety was additionally selected under the λ_min criterion, whereas it was not consistently selected in models based on imputed data. Other secondary predictors, including Age, Depression, and Frequency of pain attacks, showed variable inclusion across models.

Lasso regression models showed highly consistent results, with Physical Function, Fatigue, and Social Participation selected in nearly all models (Supplementary Table S2). Model performance metrics for elastic net and lasso models are provided in Supplementary Table S4. In the imputed data, the λ_1se elastic net model selected on average 4.30 predictors and had a cross-validated MSE of 327.1, whereas the corresponding lasso model selected 3.30 predictors and had a cross-validated MSE of 329.7. Under the λ_min criterion, performance was very similar between elastic net and lasso models.

## Discussion

In this study, we assessed preference-based HRQoL (measured by EQ-5D-5L) and PROMIS health domain scores in patients with rheumatologic diseases. As expected, both HRQoL and PROMIS domain scores were significantly lower compared to the general population. Our analyses identified Physical Function, Fatigue, and Ability to Participate in Social Roles and Activities as the most stable predictors of HRQoL. These findings underline the clinical value of PROs in capturing the patient experience.

The finding that rheumatology patients experience significantly lower HRQoL is in line with prior literature across various rheumatic diseases, including ankylosing spondylitis, psoriatic and rheumatoid arthritis, connective tissue diseases, and vasculitis [39–41]. Two PROMIS domains showed particularly large differences compared to the general population. First, the physical function domain differed by nearly 1.5 standard deviations, with a mean T-score of 36.5, close to the threshold of 35 below which patients report severe impairment [12]. This finding is in line with previous research that reported particularly low physical function in rheumatology patients compared to the general population [2, 31]. In everyday life, such reduced physical capability may affect activities that are central to independence, such as moving around outside the home, managing household tasks, or meeting the physical demands of work. Second, pain interference was also markedly elevated. This indicates that pain may substantially disrupt patients’ ability to engage in activities and roles that matter to them, for example by making it harder to maintain daily routines or plan ahead reliably. Although these domains capture distinct aspects of health, the combination of low physical function and high pain interference highlights the burden of musculoskeletal and inflammatory symptoms. Patients may experience both reduced physical capability and pain-related restrictions in daily, social, and other valued activities, which may increase reliance on support from others in everyday life.

In our comparison between the three most frequent rheumatologic diagnostic groups, we did not find statistically significant differences in HRQoL between patients with systemic sclerosis, rheumatoid arthritis, and vasculitis. Previous studies have reported differences in HRQoL across specific rheumatologic diagnoses, although findings vary depending on the diseases included and the classification strategy used. While we focused on the three most frequent ICD-based diagnostic groups, other studies have used different classification systems or focused on single disease entities. For instance, one study found that EQ-5D-5L VAS scores varied significantly across several immune-mediated inflammatory diseases, including vasculitis and connective tissue diseases. In that study, uveitis patients reported the highest HRQoL, while vasculitis patients showed the lowest [40]. In contrast, another study comparing rheumatoid arthritis and psoriatic arthritis found no significant differences in HRQoL between the two conditions [41]. Greenfield et al. also reported differences in HRQoL across systemic autoimmune rheumatic diseases, such as SLE, SSc, RA, and idiopathic inflammatory myopathies [42].

The absence of statistically significant differences in EQ-5D-5L VAS in our sample should therefore not be interpreted as evidence of comparable HRQoL across rheumatologic diseases. Rather, it may reflect limited statistical power to detect such differences due to unequal and relatively small subgroups, and these subgroup analyses should therefore be regarded as exploratory. The wide ranges of EQ-5D-5L VAS scores within the ICD-based groups further illustrate that HRQoL can vary substantially even among patients with the same principal diagnosis. This is consistent with previous findings in inflammatory rheumatic diseases, where patients with the same diagnosis showed wide variation in symptom severity and impact [4]. Diagnostic categories may therefore capture only part of the patient-perceived burden of rheumatologic disease. Patients with similar diagnoses may differ considerably in their capacity to manage everyday demands, remain engaged in important areas of life, and structure daily life reliably. Importantly, while EQ-5D-5L VAS did not differ significantly between ICD-based groups, PROMIS Pain Interference did, with higher scores in patients with RA than in patients with SSc. This suggests that global HRQoL measures may miss more specific symptom-related differences between diagnostic groups.

We used penalized regression models to identify a stable set of predictors of HRQoL. Physical Function, Fatigue, and Ability to Participate in Social Roles and Activities emerged as the most robust predictors of EQ-5D-5L VAS. In contrast, clinical variables, such as pain frequency or multimorbidity showed less stable associations. While such clinical data often inform treatment decisions, our results suggest that self-reported aspects of health, especially physical function, fatigue, and social participation, may be more closely tied to perceived HRQoL. These findings underscore the relevance of PROs for capturing health burden and may support more personalized and responsive treatment when integrated into routine care.

From a clinical perspective, the pooled model can be interpreted as a practical approximation of how differences in PROMIS scores may relate to overall health perception. Based on the pooled coefficients, a 10-point higher Physical Function score would correspond to an approximately 6-point higher EQ-5D-5L VAS, and a 10-point higher Social Participation score to an approximately 5.6-point higher EQ-5D-5L VAS. In contrast, a 10-point higher Fatigue score would correspond to an approximately 5-point lower EQ-5D-5L VAS. While these estimates should not be interpreted causally, they provide an intuitive framework for understanding the relative contribution of these domains to patients’ perceived health-related quality of life.

The associations between PROMIS domains and HRQoL were in the expected directions. Better physical function and greater ability to participate in social roles were linked to higher HRQoL, while higher fatigue was associated with lower HRQoL. Fatigue emerged as one of the most stable predictors across penalized regression models and sensitivity analyses. This finding confirms previous research identifying fatigue as a central symptom across rheumatologic conditions [16, 43, 44], and highlights its clinical importance, particularly given its frequent under-recognition in clinical practice [22]. Fatigue may limit patients’ ability to sustain daily routines, fulfil responsibilities, and participate consistently in family, social, or working life, while often remaining less visible than other disease manifestations.

### Implications for clinical management

Despite the availability of effective pharmacological treatments, many patients report unmet needs, especially regarding fatigue and physical functioning [45]. To address these issues, they must first be assessed. However, PROs are still not routinely measured in many clinical settings. Standardized tools such as the PROMIS instruments used in this study can help capture the subjective health status of patients, improve communication, and support shared decision-making [2, 46, 47].

In line with this, clinical guidelines increasingly emphasize non-pharmacological approaches for managing rheumatic diseases [24, 48]. For example, the EULAR recommendations for axial spondyloarthritis highlight the benefits of physical therapy, supported by meta-analytic evidence showing improvements in pain, function, mobility, and stiffness [49].

Our results suggest that similar attention should be paid to fatigue, which is rarely targeted directly in routine care. Evidence suggests that aerobic exercise and self-management programs can reduce fatigue in RA [50, 51], SLE [52–54], as well as in Sjögren’s syndrome [55]. Additionally, psychological interventions have been shown to improve fatigue and physical function, with studies from rheumatology and other chronic illnesses (e.g., cancer, long COVID, CFS) supporting their use [56–58]. Interventions targeting fatigue specifically may offer even greater benefits.

### Self-reports in rheumatology

In line with existing literature, our findings support the broader potential of routinely assessing PROMIS domains in rheumatology. PROs are already central to clinical trials and regulatory approval processes across a wide range of diseases, and initiatives like OMERACT have established core outcome sets for many rheumatologic conditions, often including pain, physical function, and HRQoL [59]. However, despite their established value, PROs are still underused in daily clinical practice.

### Strengths and limitations

Our study used state-of-the-art measures in a university hospital patient sample that included a relatively large proportion of rarer rheumatologic diseases. Some limitations to our study must be mentioned. First, the sample size is limited, and the patient sample was clinically heterogeneous, which is in part due to the different prevalence of the diseases as well because of the setting. Thus, generalizability of our findings may be limited and it is desirable to replicate the finding in further samples. In addition, as our sample consisted exclusively of inpatients from a university hospital, patients with more severe or complex disease courses may be overrepresented. This may limit generalizability to the broader rheumatology population, particularly outpatient populations. The cross-sectional design further precludes conclusions about temporal or causal relationships between PROMIS domains and HRQoL. Comparisons with the general population should be interpreted cautiously, particularly given differences in sample composition, including sex distribution. Exploratory diagnosis-specific comparisons were restricted to the three most frequent principal ICD-10-GM diagnosis groups. Although this avoided combining clinically distinct diagnoses into broad categories, the resulting groups remained small and unequal in size. Second, because the study is a secondary data analysis, the initial data set was not tailored towards a study that investigates predictors of HRQoL. Thus, some potentially significant clinical predictors such as the disease activity, disease duration, or the use of immunosuppressive drugs were not included in the analyses and future studies should aim to include a larger number of potential predictors. Third, the data set included missing values, which may have biased results. To address this, missing data were handled using multiple imputation by chained equations. Complete-case analyses were conducted as sensitivity analyses and supported the robustness of the main findings. The proportion of missing data for all analysis variables prior to imputation is reported in Supplementary Table S1. Lastly, we did not model potential non-linear associations between predictors and HRQoL. Given the sample size and exploratory focus, predictors were modeled linearly to avoid overparameterization. Future studies with larger samples should evaluate potential non-linear relationships.

## Conclusion

This exploratory cross-sectional study indicates that HRQoL is substantially reduced in patients with rheumatologic diseases. PROMIS domains, particularly fatigue, physical function, and Ability to Participate in Social Roles and Activities, were the most stable variables associated with HRQoL. These findings support the relevance of PROs for understanding perceived health burden in rheumatology. Implementing regular PRO assessment may help identify unmet needs and guide individualized, patient-centered interventions. Emphasizing systematic, patient-centered assessments of physical, emotional, and social health aspects can support clinicians in addressing the domains most relevant to patients’ quality of life.

## Supplementary Information

Below is the link to the electronic supplementary material.

Supplementary Material 1

## Data Availability

The datasets used and analyzed during the current study are available from the corresponding author on reasonable request.

## Abbreviations

ANOVA: Analysis of Variance
CAT: Computerized Adaptive Testing
COVID: Coronavirus Disease
CFS: Chronic Fatigue Syndrome
EULAR: The European Alliance of Associations for Rheumatology
HRQoL: Health-related Quality of Life
IRT: Item Response Theory
MAR: Missing at Random
OMERACT: Outcome Measures in Rheumatology
PROs: Patient-Reported Outcomes
PROMIS®: Patient-Reported Outcomes Measurement Information System^®^
RA: Rheumatoid Arthritis
RPS: Regional Pain Scale
SLE: Systemic Lupus Erythematosus
VAS: Visual Analogue Scale
VIF: Variance Inflation Factor
