# Supplementary material for: Understanding Health-related quality of life in rheumatologic diseases: insights from PROMIS® health domains
Source: J Patient Rep Outcomes. 2026 Jul 10;10:113. doi: 10.1186/s41687-026-01138-6 (PMC13356101; doi:10.1186/s41687-026-01138-6)
Supplement: Supplementary file 1 — Supplementary Material 1 [file 41687_2026_1138_MOESM1_ESM.docx]

**Additional file 1: Supplementary statistical analyses**

**Table S1: Proportion of missing data across analysis variables*^1^***

| Variable | Missing (n) | Missing (%) |
| --- | --- | --- |
| Pain duration | 24 | 11.3 |
| Frequency of pain attacks | 16 | 7.5 |
| Regional Pain Scale | 11 | 5.2 |
| EQ5D-5L VAS | 6 | 2.8 |
| Anxiety | 4 | 1.9 |
| Sleep Disturbance | 3 | 1.4 |
| Social Participation | 2 | 0.9 |
| Fatigue | 1 | 0.5 |
| Gender | 0 | 0.0 |
| Age | 0 | 0.0 |
| Number of diagnoses | 0 | 0.0 |
| Pain therapy (past year) | 0 | 0.0 |
| Pain Interference | 0 | 0.0 |
| Physical Function | 0 | 0.0 |
| Depression | 0 | 0.0 |
| *^1^*Percentages refer to the proportion of missing observations prior to multiple imputation. | | |

**Table S2: Selection frequency of predictors using LASSO regression*^1^***

| Predictor | Selection frequency (%) |
| --- | --- |
| Fatigue | 100 |
| Physical Function | 100 |
| Social Participation | 100 |
| Frequency of pain attacks | 25 |
| Regional Pain Scale | 5 |
| *^1^*LASSO regression (α = 1) applied to 20 imputed datasets using the 1-standard-error rule. | |

**Table S3: Average coefficient estimates across imputations*^1^***

| Predictor | Mean coefficient | SD | Selection frequency (%) |
| --- | --- | --- | --- |
| (Intercept) | 44.095 | 5.836 | 100 |
| Physical Function | 0.341 | 0.037 | 100 |
| Social Participation | 0.297 | 0.087 | 100 |
| Fatigue | -0.251 | 0.042 | 100 |
| Frequency of pain attacks | 0.200 | 0.249 | 70 |
| Regional Pain Scale | -0.007 | 0.018 | 20 |
| Depression | -0.005 | 0.016 | 20 |
| Pain Interference | -0.003 | 0.009 | 15 |
| Age | -0.002 | 0.009 | 5 |
| *^1^*Coefficients derived from elastic net models (α = 0.5) using the 1-standard-error rule. Values represent mean and SD across 20 imputations. Coefficients are penalized and should be interpreted descriptively. | | | |

**Table S4: Model performance of penalized regression models**

| Data | Regularization | Model | Cross-validated Mean Squared Error | Number of predictors |
| --- | --- | --- | --- | --- |
| Complete-case | Elastic net (α = 0.5) | λ1se | 291.9 | 3.00 |
| Complete-case | Elastic net (α = 0.5) | λmin | 257.1 | 9.00 |
| Imputed | Elastic net (α = 0.5) | λ1se | 327.1 | 4.30 |
| Imputed | Elastic net (α = 0.5) | λmin | 305.8 | 8.25 |
| Imputed | LASSO (α = 1) | λ1se | 329.7 | 3.30 |
| Imputed | LASSO (α = 1) | λmin | 305.9 | 7.80 |
| λ1se: 1-standard-error rule; λmin: minimum cross-validated error. | | | | |
| Imputed results represent averages across 20 imputed datasets. | | | | |
